# On Veratrum Viride

**Published:** 1861-04

**Authors:** 


### *On Veratrum Viride.* By Dr. Otterson.

I do not propose to read a disquisition upon the veratrum viride, but simply to call the attention of this Society to this article of the materia medica, for the purpose of opening a discussion and eliciting the experience of its members upon its use, and at the same time take the liberty of making a few suggestions for its practical application.

A few years ago, when the paper of Dr. Norwood was first published, and the article came to be prescribed and canvassed among us, many of our physicians and most of the apothecaries supposed reference was had to the ordinary white hellebore, which has long been known in our shops, and in works upon materia medica.

The veratrum viride is not the white or European hellebore, furnished by the mountainous districts of the continent; but is the American hellebore, and is found in swamps and low grounds from Maine to Georgia; it is on this account sometimes designated swamp hellebore. In the United States Pharmacopœia and Dispensatory it is called veratrum viride, and by that name it is now familiarly recognized.

This article has never been used in the regular practice to any extent, although Prof. Tully, of Yale College, New Haven, had been in the habit of prescribing it in his practice, and recommending it in his lectures, many years ago. In the August number of the *American Journal of the Medical Sciences*, 1835, Dr. Charles Osgood, of Providence, R. I., published an account of his investigations and experiments with it, his attention having been called to it by Prof. Tully; still it received little or no attention from the profession at large until the papers of Dr. Wm. C. Norwood, of Cokesbury, S. C., appeared about the year 1850.

That gentleman had been using it for several years, and his papers on the subject aroused the attention of the profession to what many practitioners, and myself among the number, considered a remedy of great importance, as well as power. The best mode of prescribing the veratrum viride is in tincture; and as Dr. Norwood's preparation is now in the market, for the sake of uniformity, and while it is comparatively a new remedy, I should recommend that it be always prescribed. Its effects upon the system are generally prompt, certain and of such power as not to be entirely void of danger. In its operation as well as in its nativity, the veratrum viride differs from the ver. alba., in that it does *not*, like the alb., produce catharsis, which is the principal operation of the latter; on the contrary, it is quite promptly emetic, and not cathartic at all. But its chief action, and that which gives to it its remedial value, is its wonderful control over the circulatory and nervous systems. It possesses in a preëminent degree the power of calming nervous excitement, and reducing the force and frequency of the heart's contractions.

It is indicated in all that class of sthenic diseases where prompt and persistent sedatives are required; and has the advantage over the lancet, in that it can be continued by slight repetitions, without reducing the vital powers, as would the same repetition of the lancet. It is preferable to digitalis, in that it is more prompt and certain, and has not yet been found to possess that cumulative property which constitutes one of the dangers of, and objections to, digitalis. It is preferable to antimony, in that it does not produce such violent enteritic irritation and griping diarrhœa, while as a sedative it is considered by many superior; and as an *expectorant*, fully equal to the antimony; its diaphoretic properties are about the same; in its local effects, it somewhat resembles this drug, as, when applied to the skin, it produces heat and irritation, though not such an eruption as will the antimony. The same may be said of its effects upon the alimentary canal. Hence it is contra-indicated when any gastric or enteritic irritation exists. I will now, in as few words as possible, introduce this new friend to a place in the society of those old and long-tried ones, upon whose virtues we have been so long accustomed to rely. Nor have I any doubt that the addition will be found both pleasant and profitable.

I would recommend Norwood's tincture to be prescribed, in preference to Tilden's, or any other fluid extract, because I think it likely to be more uniform in strength, and hence its effects.

It is adapted to all diseases requiring the action of the heart and arteries to be controlled, except, as above stated, those attended by gastro-enteritic irritation.

In the latter stages of disease, where the rapid action of the heart is consequent upon the progress of the disease, it should not be employed; such as the latter stages of fevers, when there are nervous symptoms and excessive cardiac action, attended with much prostration.

I think the doses recommended by Dr. Norwood, and some others, too large to commence with, particularly by those unaccustomed to prescribing it, and would rather advise them to commence with half the quantity, carefully observing its effects until they become familiar with them, and their own experience will be their most reliable counselor.

I have used it myself in pneumonia, pleuritis, acute rheumatism, inflammatory croup, iritis, puerperal peritionitis, and nervous palpitation of the heart, and have occasionally added a few drops to expectorant mixtures, where the cough was produced or attended by bronchial irritation, which seems to be extended to the minute vesicles, and the expectoration difficult and albuminous in its character; and I must say, I consider it a most excellent addition; in its administration I have seldom commenced a case with more than three to four drops, repeating it in two hours, and then adding by one drop, at each repetition, or diminishing the quantities, and lengthening or shortening the interval as circumstances might demand. I have generally found a much less quantity to bring down the pulse as low as I required, than that mentioned by Norwood, and some others. When emesis is once produced, the pulse generally comes down sufficiently, the skin softens, or a profuse perspiration breaks out, and the great point is attained. This, as before stated, is easily continued by the occasional repetition of the remedy in very much diminished doses, varying, of course, as with all other medicines, according to the susceptibility of the patient. When emesis is once produced, I find two or three drops every hour or second hour control the symptoms which indicated its administration.

Reference has been made to the *power* of the veratrum viride. It sometimes occurs that its operation is active beyond anticipation, and excessive emesis and prostration, even it may be to an alarming degree, are produced. This may be immediately counteracted by the administration of stimulants; a little weak brandy and water with a few drops of laudanum in it, or a few spoonsful of camph. julep with laudanum, answer the purpose. I have never seen a case requiring carb. ammon., although it is advised. Sinapisms to the stomach are useful addenda to the treatment. In excessive emesis anodynes seem to be the antidote, and laudanum or syrup morphia are always at hand.

I have made this imperfect and disjointed sketch, as at first stated, in order to elicit the experience of such as may have prescribed this remedy, and with a view to bring it to the attention of those who have not. Not that I consider our pharmacopœias deficient in the number of remedies they present; by no means; and yet I think new remedies of undoubted virtues should find there a place; notwithstanding they may be new to the great majority of our members. I presume the administration of this is new. At the South it is in extensive use, and highly prized, and by those who pretend to prescribe vegetable remedies only; it is at once in combination, their lancet, their digitalis, and their antimony; with them it is *tria juncta in uno.*

While I hope I may not be accused of being carried away with a *new* thing, and making of it a hobby, which I am very positive I do not, I would yet recommend, as thousands have done before me, in every pursuit of life, that good old Scripture doctrine, "Try all things, hold fast that which is good."—*American Medical Monthly.*

